# Shear wave speed changes in the cervix and vulvar lips of Kivircik ewes before and after parturition

**DOI:** 10.3389/fvets.2025.1703721

**Published:** 2026-01-09

**Authors:** Zeynep Günay Uçmak, Melih Uçmak, İbrahim Kurban, Mehmet Fatih Özbezek, Bülent Ekiz

**Affiliations:** 1 Department of Obstetrics and Gynaecology, Faculty of Veterinary Medicine, Istanbul University-Cerrahpaşa, Istanbul, Türkiye; 2 Equine and Training Program, Vocational School of Veterinary Medicine, Istanbul University-Cerrahpaşa, Istanbul, Türkiye; 3 Institute of Graduate Studies, Istanbul University-Cerrahpaşa, Istanbul, Türkiye; 4 Department of Animal Breeding and Husbandry, Faculty of Veterinary Medicine, Istanbul University-Cerrahpaşa, Istanbul, Türkiye

**Keywords:** cervix, ewes, parturition, shear wave speed, vulvar lips

## Abstract

**Introduction:**

In recent years, the evaluation of the cervix in pregnant women using shear wave elastography (SWE) has gained attention. However, there is limited information on its use in animals. Pregnancy monitoring in sheep can reduce both maternal and lamb mortality rates. Quantitative and objective evaluation of the cervix and vulvar lips in pregnant ewes may help determine the presence of a healthy pregnancy and the timing of parturition. This study aimed to evaluate the changes in cervical diameter, vulvar thickness, and shear wave speed (SWS) of the cervix and vulvar lips during the days preceding normal parturition and on the day of postpartum in Kivircik ewes.

**Methods:**

Ultrasonographic measurements of the 12 ewes included in the study were taken daily, starting 5 days before expected parturition and continuing until 6–12 h postpartum (Day 0). The SWS of the cervix was measured in two different regions: near the internal os of the cervix (IOC) and near the external os of the cervix (EOC). Time-dependent differences in the evaluated cervical and vulvar parameters were assessed using the general linear model and repeated measures ANOVA. When statistical significance was detected, the LSD pairwise comparison test was used to determine the significance of differences in the investigated parameters across the evaluation days.

**Results:**

The highest cervical diameter was recorded on Day −1. Time-dependent differences in mean SWS values were significant for the IOC but insignificant for the EOC. The mean SWS value of the IOC on Day −1 (2.29 ± 0.13 m/s) was lower than the values on all other evaluation days. The mean SWS value of the IOC on Day 0 was higher than the values on Days −1, −2, and −3. The thickness of the vulvar lips on Day −1 was similar to that on Day 0, but both were higher than the thickness measured on all other evaluation days. The mean SWS value of the vulvar lips on Day −1 was similar to those on Days −2 and 0, but it was lower than the value on Days −5, −4, and −3.

**Conclusion:**

In conclusion, it was possible to identify changes in the cervix and vulvar lips during the days preceding normal parturition and on the day of postpartum in ewes. Final ripening of the IOC and edema of the vulvar lips were reflected by decreased SWS values. Marked uterine contractions persisting during the postpartum period resulted in increased SWS values at the IOC.

## Introduction

Ewe and lamb mortality is a major economic problem in the sheep industry. Most deaths occur during the peri-parturient period and are associated with dystocia (1). Infectious or non-infectious abortions are also a problem for sheep (2). The main causes of dystocia are fetopelvic disproportion, uterine inertia, failure of the cervix to fully dilate, malpresentation, and congenital defects in lambs (1). Incomplete dilatation of the cervix, a condition known as ringwomb, is responsible for 15–32% of dystocia cases. As parturition approaches, some alterations occur in the cervix due to mechanical, hormonal, neural, and biochemical mechanisms, which lead to its softening and relaxation (3). Appropriate and timely cervical remodeling is essential for successful parturition. Hormonal imbalances in the fetal hypothalamic–pituitary–adrenal axis may cause inadequate dilation of the cervix. Environmental stress factors are also associated with ringwomb (1). Predicting the time of parturition allows farmers to ensure that their sheep have successful parturition and reduces both maternal and lamb mortality rates in cases of dystocia (4). In very early studies, the timing of parturition was estimated based on changes in the rectal temperature of the sheep (5). In recent years, pregnancy can be monitored with rapidly advancing technologies (6).

New developments in ultrasound technology have enabled elastography-based imaging and assessment techniques. Tissue elasticity refers to the tendency of a tissue to resist deformation when a force is applied, or to return to its original shape after the removal of the force. Ultrasound elastography techniques are divided into two main methods: Strain and shear wave elastography (SWE). In strain or static elastography, tissue displacement under compression is measured. Compression is applied manually by the operator pressing the ultrasound transducer against the tissue. In SWE (dynamic elastography), the propagation speed of a shear wave induced by an ultrasound probe is determined. Using SWE, a color-coded elastogram (ranging from dark blue to red) is obtained, and shear wave speed (SWS; m/s) is measured at the desired region of interest (ROI) (6–9). Shear waves travel faster in stiffer tissues and slower in softer tissues (10). The main clinical applications of SWE have been in assessing human liver fibrosis and breast lesions in women (7–9). In addition, the evaluation of cervical tissue elasticity using the SWE technique is also employed in human medicine during healthy pregnancy (11–15) and in cases of preterm birth (16).

A firm and closed cervix is essential for the continuation of pregnancy. The portion of the cervical canal that opens into the uterine cavity is called the internal os of the cervix (IOC), and the portion that opens into the vagina is called the external os of the cervix (EOC) (15, 17). During pregnancy, the cervix undergoes four different phases: initial softening, ripening, active dilation aided by uterine contractions, and postpartum recovery (15, 18). Cervical stiffness decreases significantly with gestational age in women, as indicated by lower kPa (11) and SWS values (12–14). There are limited studies on the application of SWE in sheep reproduction. Only two studies evaluating the sheep cervix using SWE have been identified. In the first study, cervical stiffness was evaluated during ripening induced by dexamethasone injection (19). The second study is our previous report, which assessed the cervix during the postpartum period in Kivircik ewes (20). Other studies using elastography in ewes have focused on gestational monitoring of fetal structures and placentomes (21), as well as on uterine wall assessment during the postpartum period (22).

This study aimed to (1) evaluate changes in the SWS of the cervix and vulvar lips of Kivircik ewes during the days preceding normal parturition and on the day of postpartum, (2) evaluate changes in cervical diameter and vulvar lip thickness at the evaluation points, (3) compare the SWS values of the IOC and EOC, and (4) investigate correlations among the parameters at the evaluation points.

To the best of our knowledge, this is the first study to use SWE to evaluate the cervix and vulvar lips during the days preceding normal parturition in ewes.

## Materials and methods

This study was approved by the Unit Ethics Committee of Istanbul University-Cerrahpaşa, Faculty of Veterinary Medicine, Istanbul, Türkiye (Approval No. 2023/20).

### Animals and study design

The study was carried out at the Istanbul University-Cerrahpaşa Faculty of Veterinary Medicine Sheep Farm, located at 40°59′19.3″N, during the parturition season (March to May). Ultrasonographic examinations were initiated on ewes whose proximity to parturition was estimated using the farm records containing information about mating days. A total of 12 ewes with complete data sets for at least 5 days before parturition were included in the study. All ewes (nine with singleton pregnancies and three with twin pregnancies) were 2–4 years old, had a body weight of 55–60 kg, and underwent normal, unassisted parturition. Balanced concentrate feed, ad libitum quality alfalfa hay, and fresh water were provided to the ewes. Ultrasonographic measurements were taken daily, starting 5 days before the expected parturition (Day −5, Day −4, Day −3, Day −2, Day −1) and continuing until 6–12 h postpartum (Day 0).

### Ultrasound examinations

**Figure 1:**
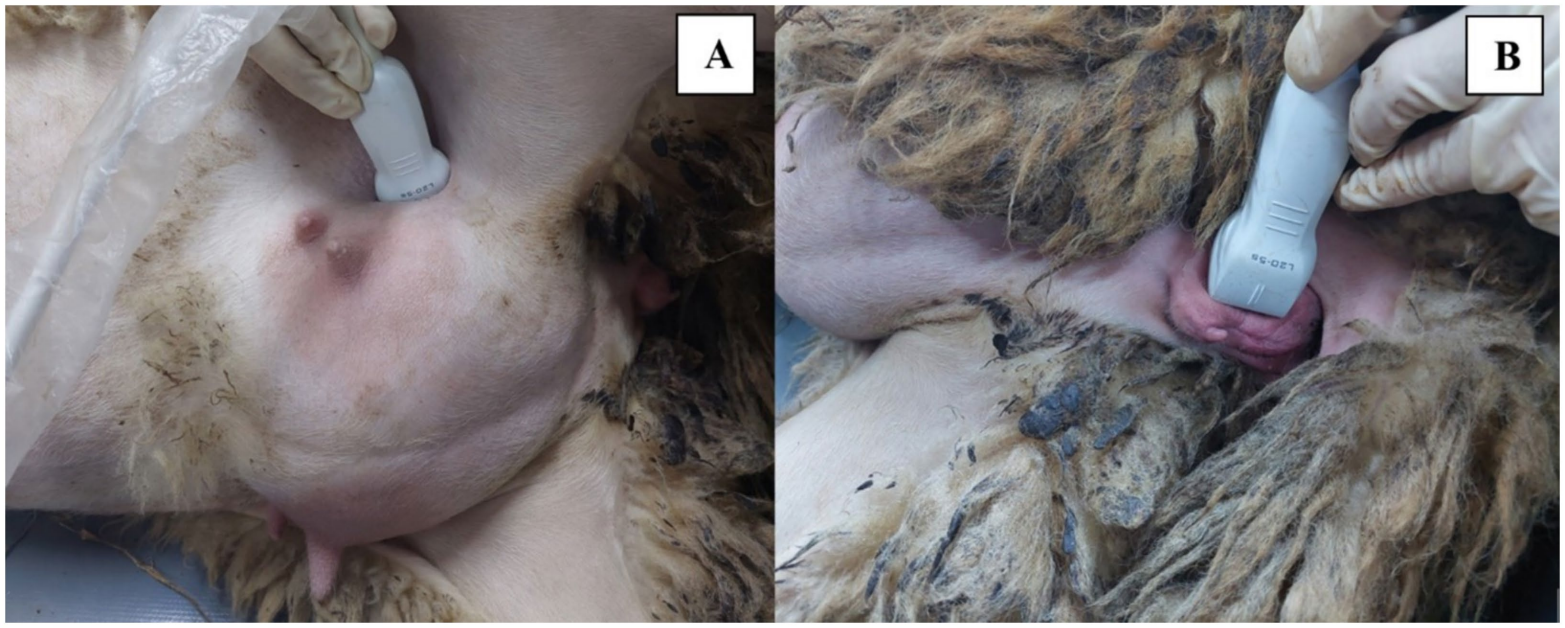
Posture of the sheep and positioning of the transducer during ultrasound examination. **(A)** Visualization of the cervix cranial to the mammary glands on the ventrolateral abdomen. **(B)** Evaluation of the vulvar lips with a vertically positioned transducer.

All measurements were carried out by the same clinician (ZGU). Scanning was performed transabdominally using a Mindray Resona i9 ultrasound system (Mindray, China) with a linear transducer (L20-5 s). All ewes were positioned on their right side. The abdominal–inguinal area was clipped to facilitate ultrasound assessment, and ultrasound gel was applied to eliminate air pockets. The cervix was visualized cranial to the mammary glands on the ventrolateral abdomen in a longitudinal view (Figure 1A). Due to advanced pregnancy, the cervix could be easily visualized from this area. In addition, the same transducer was placed on each vulvar lip for evaluation (Figure 1B). The widest cross-sectional diameter of the cervix and the mean thickness of the vulvar lips were measured using B-mode ultrasonography.

### Shear wave elastography

The settings during elastography examination were as follows: HQE off, quality penetration (Q Pen) frequency, Map E2, opacity (OP) set to 4, iLay off, and filter set to 1 for all evaluated tissues. Qualitative and quantitative image analyses were performed for elastography measurements. During elastograpy measurements, the motion stability index (M-STB index) and the reliability index (RI) were taken into consideration. Motion stability index (M-STB index) ≥4 consecutive green stars indicated the images were captured in a stable state and were considered valid measurements. The reliability index (RI) ≥ 85% was considered to indicate high image quality. The cervix was evaluated separately in two different regions. To determine the SWS of the cervix in the longitudinal view, two ROIs were placed near the IOC, corresponding to the cranial portion of the cervix, and two ROIs were placed near the EOC, corresponding to the caudal portion of the cervix. In addition, a total of four ROIs were placed on the vulvar lips (two on each lip). The ROI placement within the elastography image for SWS was determined solely based on anatomical criteria, and all measurements were performed in avascular areas of the relevant tissues (previously checked by a power Doppler scan). For the quantification of SWS (m/s), the ROI was determined as circular, and its diameter was kept constant at 2 mm for each tissue. The mean, minimum, and maximum SWS values were automatically displayed by the system, and only the mean SWS values were used. The mean value of the two ROI measurements was used to determine the SWS values of the IOC and EOC, and the mean value of the four ROI measurements was used to determine the SWS values of the vulvar lips.

### Statistical analysis

Data were analyzed using the statistical software SPSS 23.0 (SPSS Inc., Chicago, IL, United States). The normality of the data was assessed using the Shapiro–Wilk test. Time-dependent differences in the evaluated parameters of the cervix and vulvar lips were assessed using the general linear model and repeated measures ANOVA. When statistical significance was detected, the LSD pairwise comparison test was used to determine the significance of differences in the investigated parameters across the evaluation days. Student’s *t*-test was used to compare the SWS values of the IOC and EOC and to test for any significant differences between the singleton and twin pregnancies. Associations among the evaluated parameters (diameter, thickness, and SWS) in the respective tissues on the evaluation days were assessed using the Pearson correlation coefficient. Significance was set at a *p*-value of <0.05. The results were expressed as mean ± standard error and were also illustrated using box plots. The “x” signs in the boxes represent the mean values, the interior lines represent the medians, the edges of the boxes are the upper and lower quartiles (25th and 75th percentiles), the bars (‘whiskers’) represent the maximum and minimum values, and the circular markers are outliers.

## Results

All ewes had successful parturition and expelled their placentas within 6 h of delivery. A total of nine of the 12 ewes had singleton offspring, while the remaining three had twins. The number of fetuses per ewe did not affect any of the ultrasonographic measurements at any evaluation time (*p* > 0.05). Ultrasonographic measurements taken from each sheep from Day-5 to Day 0 were used in the study.

### Cervix

**Figure 2:**
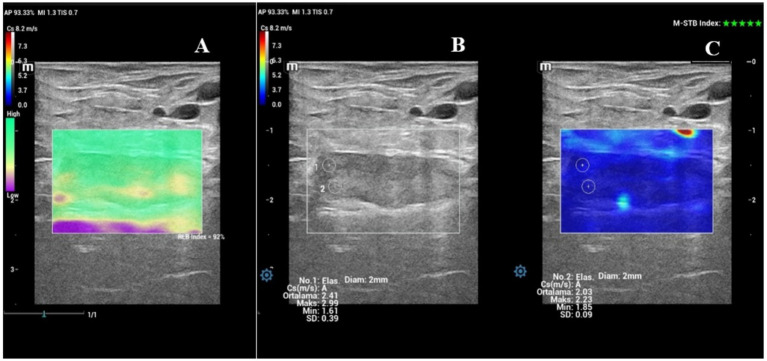
Shear wave elastography of the internal os of the cervix (IOC) on Day −1. **(A)** Shear wave image of the IOC with a 92% RLB index. **(B)** Quantitative evaluation of the IOC with two ROIs; M-STB index: 5 stars. **(C)** Qualitative map of shear wave elastography of the IOC.

**Figure 3:**
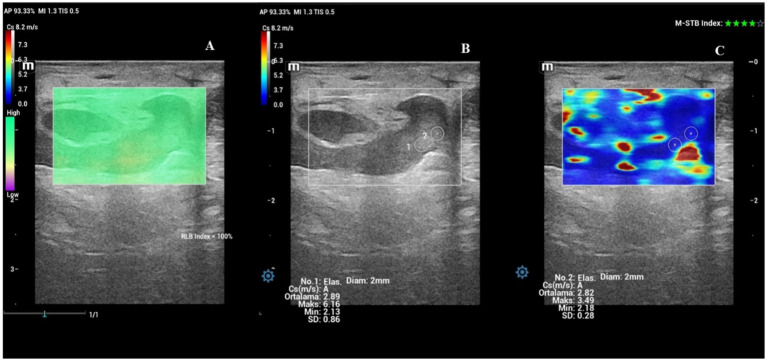
Shear wave elastography of the external os of the cervix (EOC) on Day −1. **(A)** Shear wave image of the EOC with a 100% RLB index. **(B)** Quantitative evaluation of the EOC with two ROIs; M-STB index: 4 stars. **(C)** Qualitative map of shear wave elastography of the EOC.

**Figure 4:**
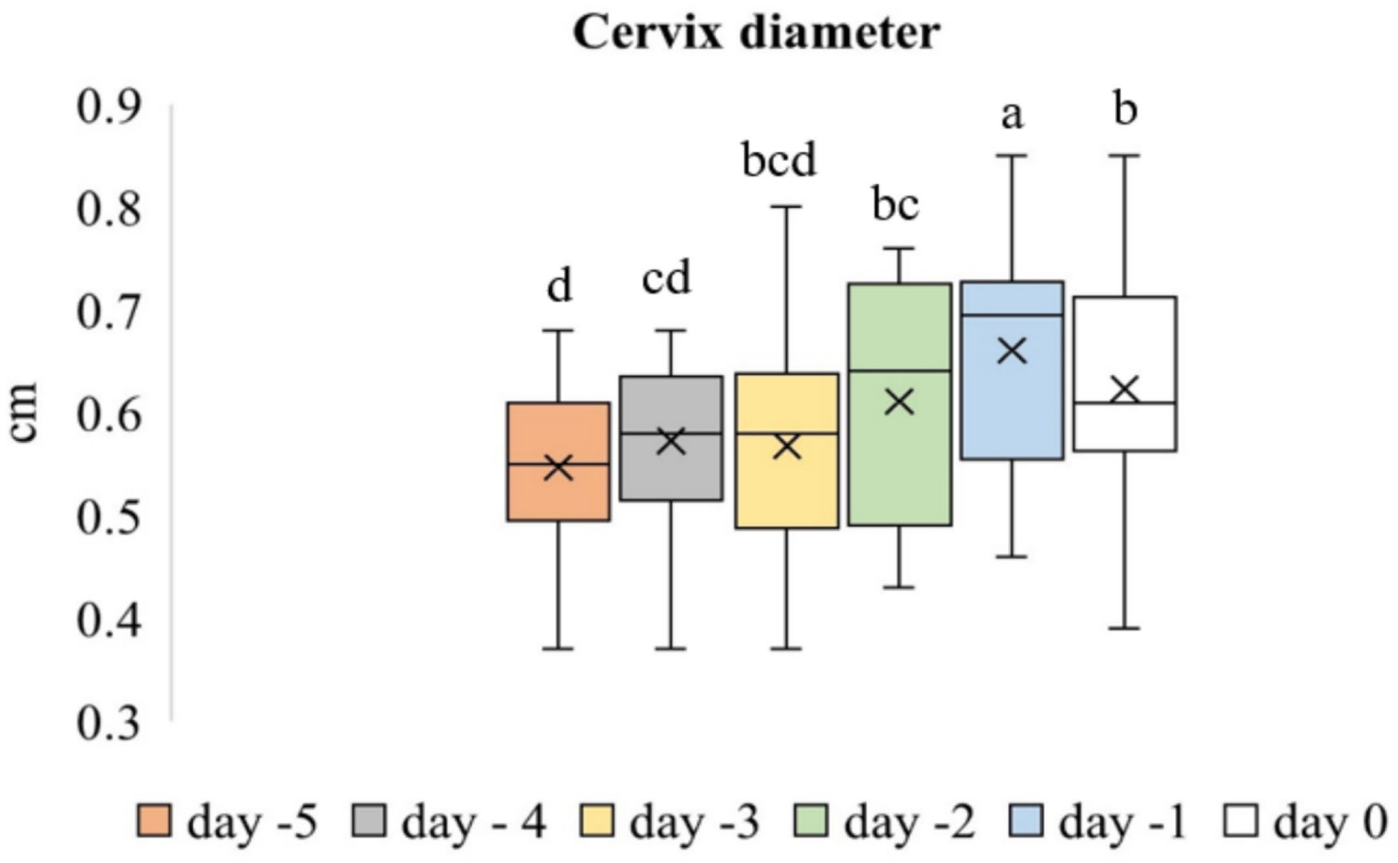
Box plot analysis of cervical diameter across the evaluation days. The “x” signs in the boxes represent the mean values, the interior lines represent the medians, the edges of the boxes are the upper and lower quartiles (25th and 75th percentiles), and the bars (‘whiskers’) represent the maximum and minimum values. Values that do not share the same letter are significantly different (*p* < 0.05).

The evaluation of the cervix included the widest diameter (cm) and SWS values of the IOC (Figures 2A–C) and EOC (Figures 3A–C), which were measured in the longitudinal view. Time-dependent differences in the diameter of the cervix were significant (*p* < 0.001). Figure 4 shows the box plot analysis of cervical diameter and the differences over the evaluation days. The highest cervical diameter was recorded on Day −1, with a mean value of 0.66 ± 0.03 cm. Cervical diameter on Day −1 was higher than that on Days −5 (*p* < 0.001), −4 (*p* < 0.001), −3 (*p* < 0.05), and −2 (*p* < 0.05).

**Figure 5:**
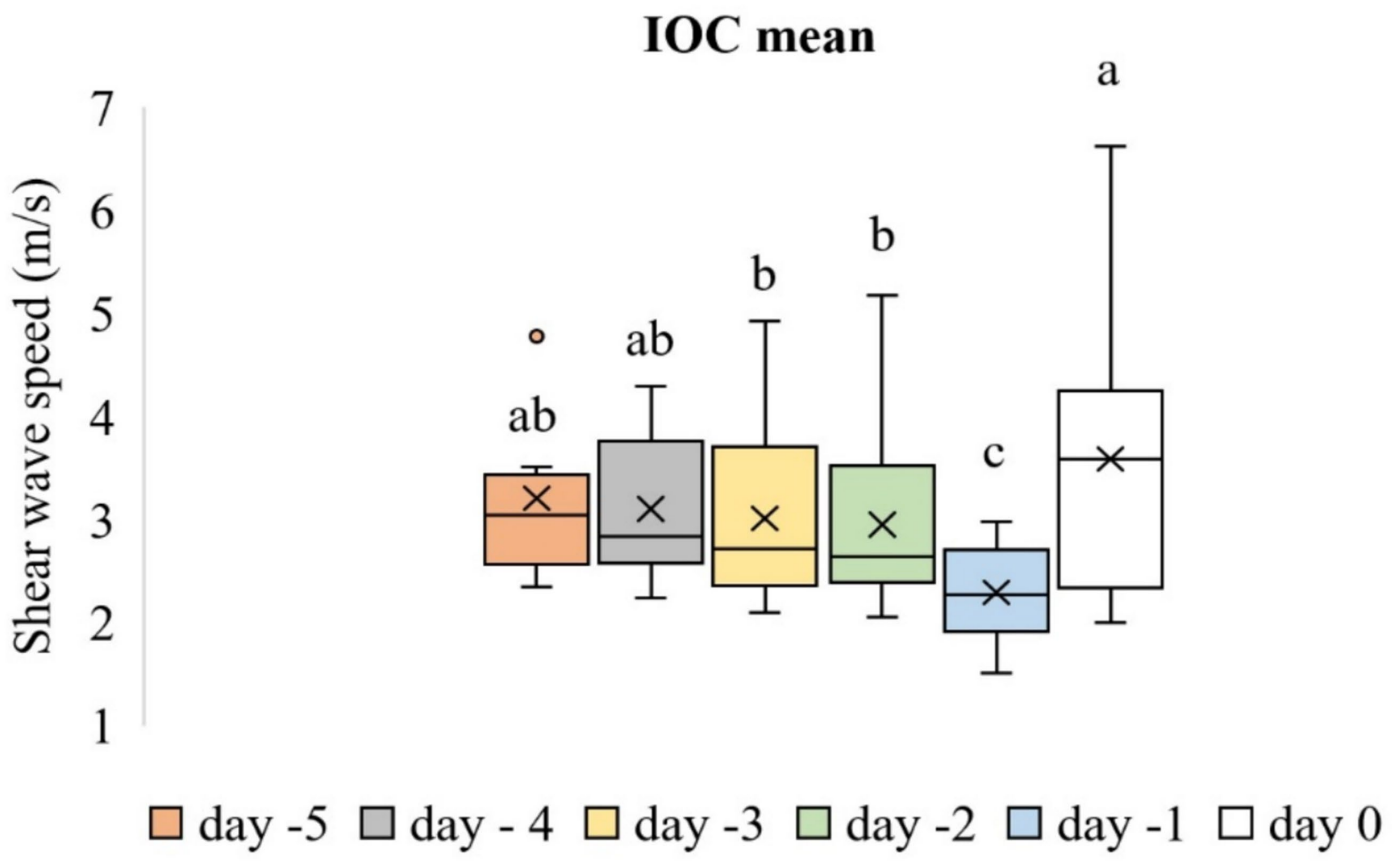
Box plot analysis of the mean SWS values of the IOC across the evaluation days. The “x” signs in the boxes represent the mean values, the interior lines represent the medians, the edges of the boxes are the upper and lower quartiles (25th and 75th percentiles), the bars (‘whiskers’) represent the maximum and minimum values, and the circular markers are outliers. Values that do not share the same letter are significantly different (*p* < 0.05).

**Figure 6:**
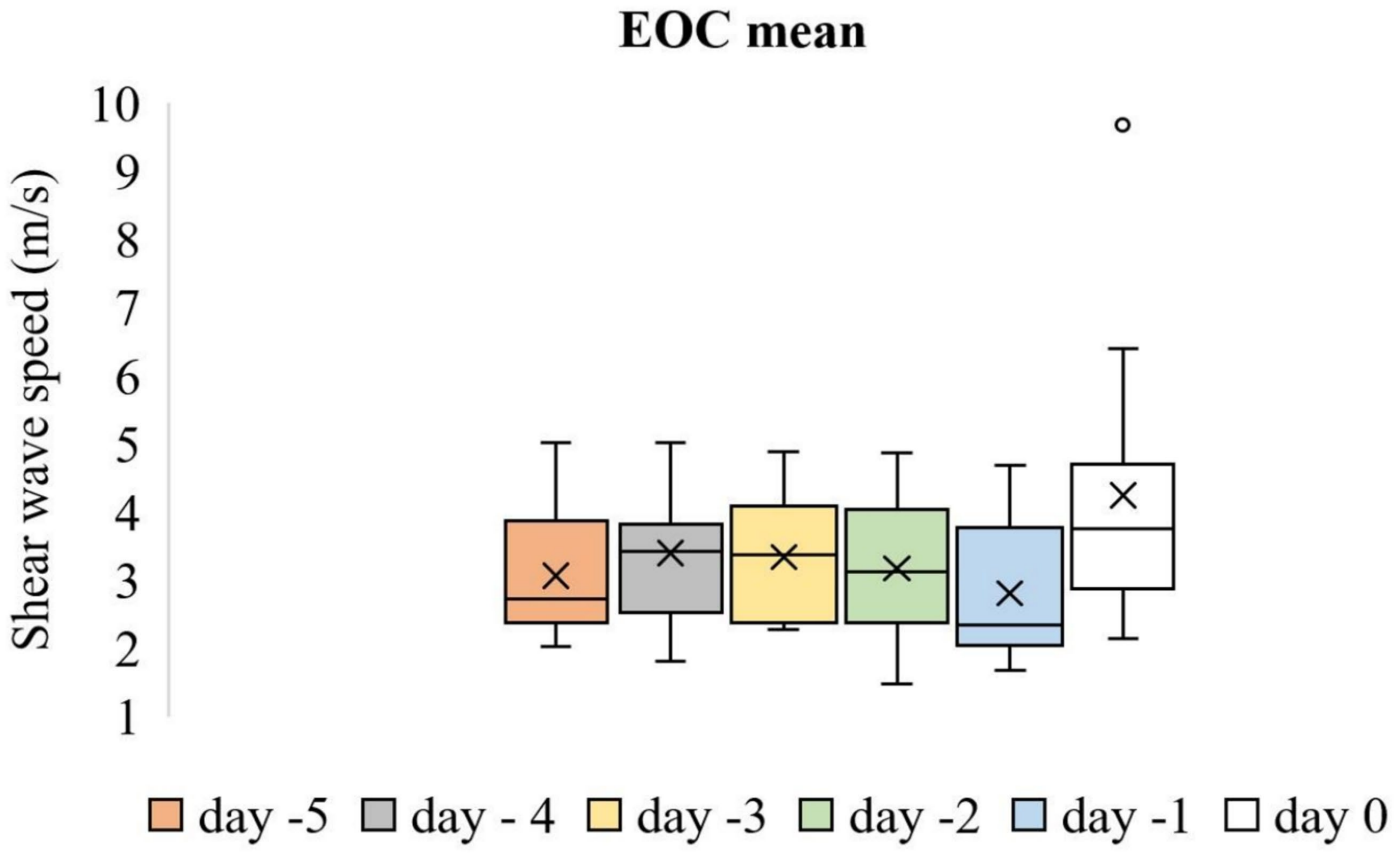
Box plot analysis of the mean SWS values of the EOC across the evaluation days. The “x” signs in the boxes represent the mean values, the interior lines represent the medians, the edges of the boxes are the upper and lower quartiles (25th and 75th percentiles), the bars (‘whiskers’) represent the maximum and minimum values, and the circular markers are outliers.

Time-dependent differences in the mean SWS values of the IOC were significant (*p* < 0.001). Figure 5 shows the box plot analysis of the mean SWS values of the IOC and the differences across the evaluation days. The mean SWS value of the IOC on Day −1 (2.29 ± 0.13 m/s) was lower than that on all other evaluation days (*p* < 0.05). However, time-dependent differences in the mean SWS values of the EOC were not significant (*p* > 0.05). Figure 6 shows the box plot analysis of the mean SWS values of the EOC across the evaluation days. The mean SWS values of the IOC and EOC are compared in Table 1.

**Table 1:** Comparison of the mean SWS values of the IOC and EOC across the evaluation days.

Regions of cervix	Day −5	Day −4	Day −3	Day −2	Day −1	Day 0
IOC (m/s)	3.21 ± 0.27	3.10 ± 0.20	3.01 ± 0.25	2.95 ± 0.26	2.29 ± 0.13	3.59 ± 0.37
EOC (m/s)	3.06 ± 0.29	3.40 ± 0.28	3.34 ± 0.26	3.18 ± 0.30	2.80 ± 0.28	4.25 ± 0.59
P-value	ns	ns	ns	ns	<0.05	ns

The values are presented as mean ± standard error.

Furthermore, correlations among the parameters at the evaluation points were investigated. On Day 0, the diameter of the cervix was inversely correlated with the mean SWS value of the EOC (*r* = −0.58). The mean SWS values of the IOC and EOC on Days −3, −2, and −1 were positively correlated (*r* = 0.66, *r* = 0.71, r = 0.64, respectively).

### Vulvar lips

**Figure 7:**
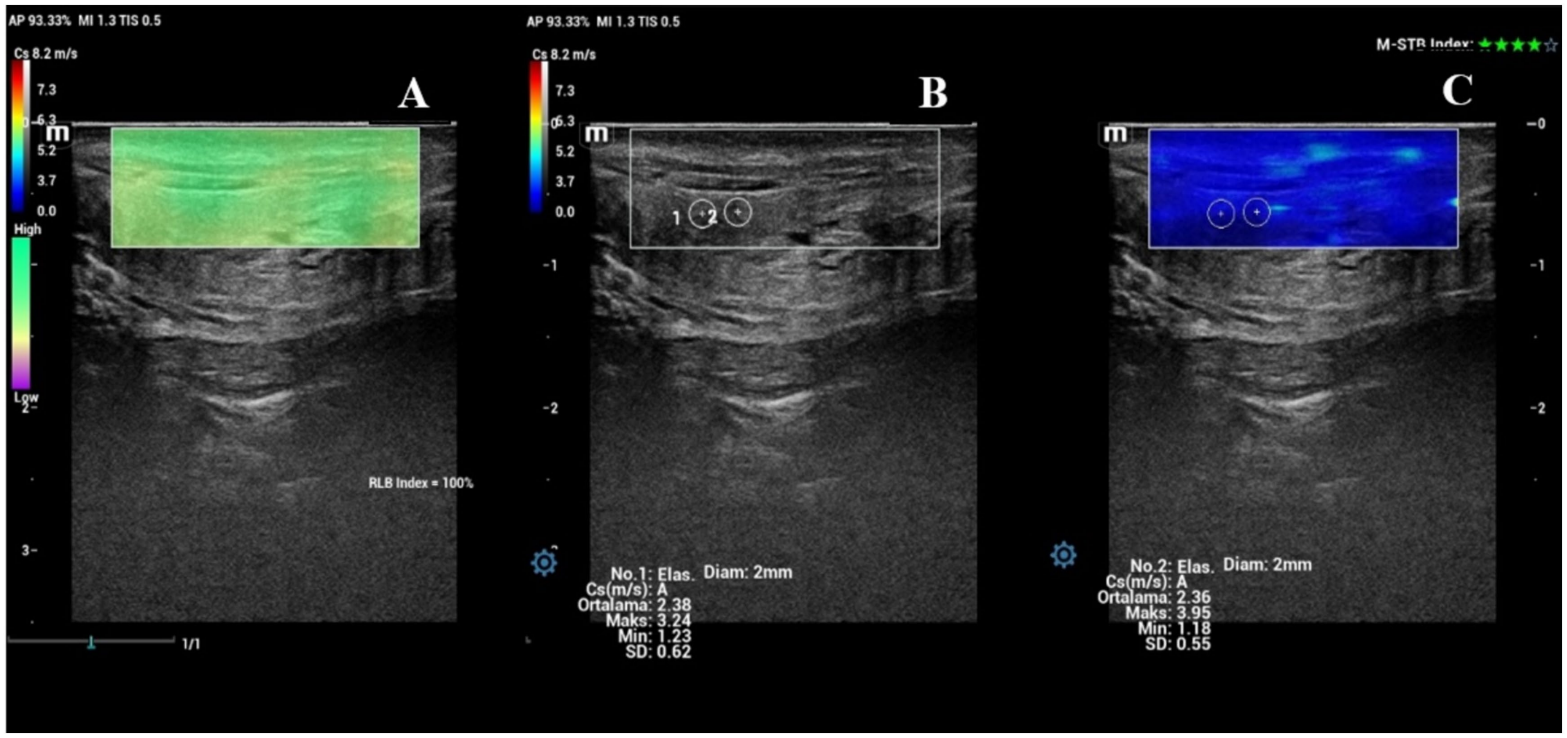
Shear wave elastography of a vulvar lip on Day −1. **(A)** Shear wave image of the vulvar lip with a 100% RLB index. **(B)** Quantitative evaluation of the vulvar lip with two ROIs; M-STB index: 4 stars. **(C)** Qualitative map of shear wave elastography of the vulvar lip.

**Figure 8:**
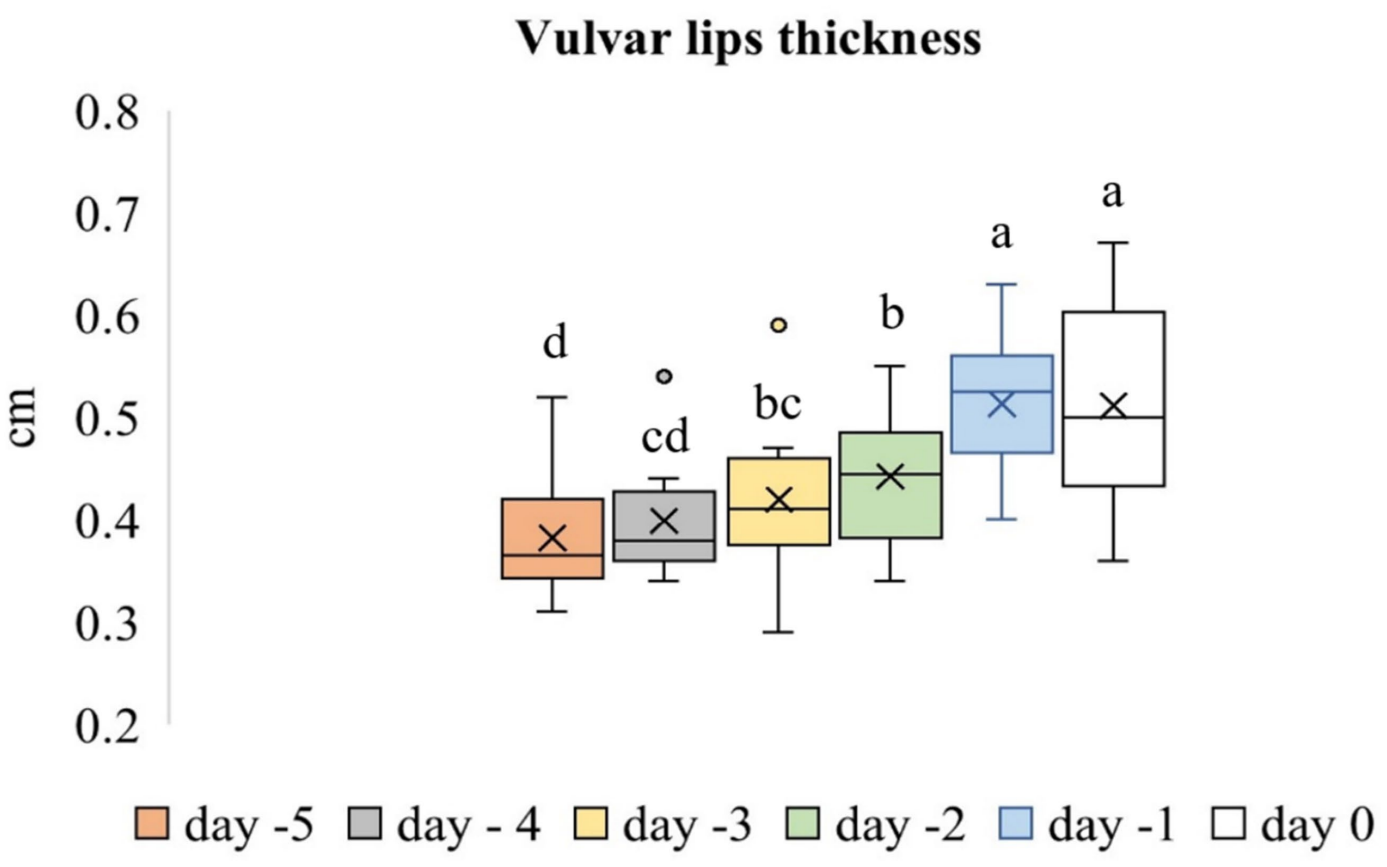
Box plot analysis of vulvar lip thickness across the evaluation days. The “x” signs in the boxes represent the mean values, the interior lines represent the medians, the edges of the boxes are the upper and lower quartiles (25th and 75th percentiles), the bars (‘whiskers’) represent the maximum and minimum values, and the circular markers are outliers. Values that do not share the same letter are significantly different (*p* < 0.05).

**Figure 9:**
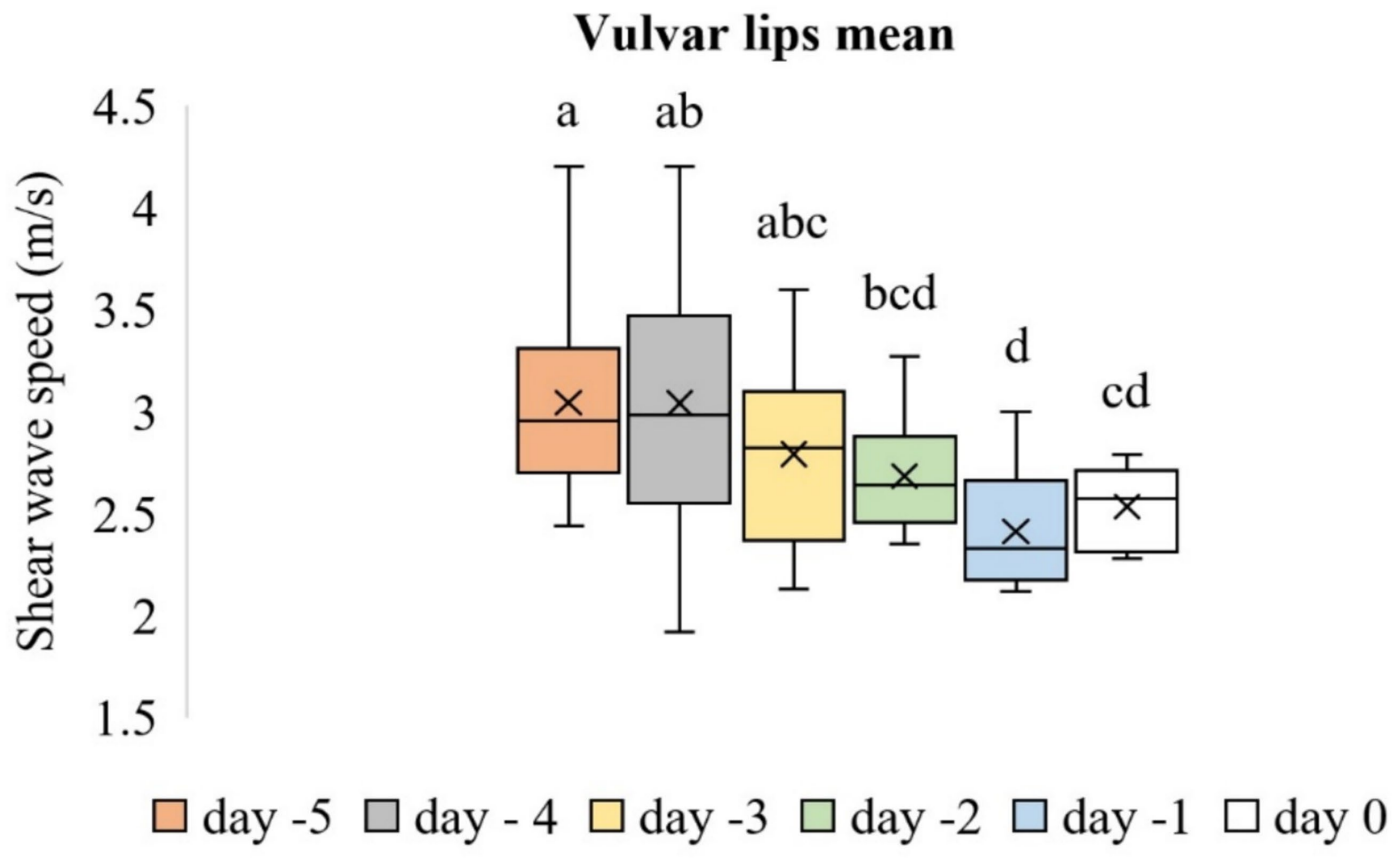
Box plot analysis of the mean SWS values of the vulvar lips across the evaluation days. The “x” signs in the boxes represent the mean values, the interior lines represent the medians, the edges of the boxes are the upper and lower quartiles (25th and 75th percentiles), and the bars (‘whiskers’) represent the maximum and minimum values. Values that do not share the same letter are significantly different (*p* < 0.05).

The evaluation of the vulvar lips included thickness and SWS values (Figures 7A–C). Time-dependent differences in the thickness of the vulvar lips were significant (*p* < 0.001). The thickness of the vulvar lips on Day −1 was similar to that on Day 0 (*p* > 0.05), but both were greater than the thickness measured on all other evaluation days (*p* < 0.05). Figure 8 shows the box plot analysis of the thickness of the vulvar lips and the differences over the evaluation days. Time-dependent differences in the mean SWS values of the vulvar lips were significant (*p* < 0.001). Figure 9 shows the box plot analysis of the mean SWS values of the vulvar lips and the differences across the evaluation days. The mean SWS value of the vulvar lips on Day −1 was similar to the values on Days −2 and 0 (*p* > 0.05), but it was lower than the values on Days −5, −4, and −3 (*p* < 0.05).

## Discussion

With the development of SWE techniques in recent years, many studies have evaluated female reproductive tissues during pregnancy (10, 17). However, the use of SWE in sheep reproduction is still limited (19–22). In this study, we evaluated changes in cervical diameter, vulvar thickness, and the SWS of the cervix and vulvar lips during the days preceding normal parturition and on the day of postpartum in Kivircik ewes. To the best of our knowledge, this is the first study conducted on this subject in ewes.

da Silva et al. (21) used acoustic radiation force impulse elastography to assess maternal structures and placentomes in pregnant ewes. In line with their findings (21), we also did not observe any adverse effects of SWE on either the dam or the lamb, as indicated by the absence of mortality, dystocia, or congenital abnormalities detectable on physical examination.

In sheep, the softened cervix remains long and closed throughout pregnancy until 12 h prior to uterine contractions of labor (23). Cervical dilation usually begins 5 h before normal parturition (24). In this study, although the cervix evaluated on Day −1 was not yet in the dilation phase, its diameter was larger than that at previous evaluations. This may be related to changes in hormonal concentrations (estrogen/progesterone) and cervical tissue composition that occur in the period close to parturition. The diameter of the cervix obtained on Day −1 was also greater than that measured on Day 0. Wehrend et al. (25) emphasized the importance of the closure of the cervix immediately after expulsion for the protection of the cavum uteri, and they evaluated the reduction process of the cervix in four phases, with the first phase being the fastest and lasting up to 16th h postpartum. Mariano et al. (22) observed a remarkable reduction in uterine diameter during the first day postpartum. In this study, the Day 0 evaluation conducted 6–12 h after delivery supports the rapid recovery process reported in previous studies (22, 25).

Carlson et al. (26) first evaluated the SWS of the human pregnant cervix in an *in vivo* study. They detected an increase in softening (a decrease in SWS values) from the pre- to post-ripening cervix; 2.53 ± 0.75 and 1.54 ± 0.31 m/s, respectively. Researchers also quantified SWS in the human cervix throughout pregnancy (12–14). Horinouchi et al. (12) studied 362 women at 12–35 weeks of gestation and evaluated SWS in different cervical regions, finding that SWS decreased over time, confirming the gradual softening of the cervix throughout pregnancy. Ono et al. (15) found significant negative correlations between stiffness and women’s gestational age. Carlson et al. (14) found higher SWS values in the first trimester of pregnancy compared to the third trimester (4.42 ± 0.32 m/s and 2.13 ± 0.66 m/s, respectively), thereby demonstrating cervical softening throughout pregnancy. In an animal model study conducted with Rhesus macaque (27), the sensitivity of SWS to cervical softening was demonstrated, and the SWS of the cervix was found to decrease with pregnancy. Peralta et al. (19) evaluated cervical stiffness in ewes at day 127 of pregnancy during induced ripening following dexamethasone injection. After 24 h, SWS decreased from 1.779 ± 0.548 to 1.291 ± 0.516 m/s and stiffness from 9.5 ± 0.9 kPa to 5.0 ± 0.8 kPa.

In this study, we evaluated the SWS of the cervix in Kivircik ewes in two different regions, the IOC and EOC, during the days preceding normal parturition and on the day of postpartum. Time-dependent differences in the mean SWS values of the IOC were significant, and the mean SWS value of the IOC on Day −1 (2.29 ± 0.13 m/s) was lower than that on all other evaluation days. The lower SWS value obtained on Day −1 means softer cervical tissue, which could be related to the accelerated ripening process before normal parturition in ewes. This decrease in the mean SWS value of the IOC on Day −1 is consistent with reports in women (26). In this study, the reduction in the mean SWS value on Day −1 of normal parturition is also consistent with the decrease reported in induced lambing in ewes (19). Although the decreasing trend in SWS was similar in both studies, the relatively higher SWS values in this study may be due to the use of sedation or an endocavitary ultrasonic probe by Peralta et al. (19).

The cervix is composed of fibrous connective tissue (collagen and elastin fibers). Hyaluronic acid is related to the capacity of a tissue to imbibe fluid. Cervical changes during pregnancy occur due to two complementary factors: collagen degradation by collagenase enzymes and changes in the relative amount of aminoglycans (an increased concentration of hyaluronic acid and a decreased concentration of dermatan sulfate) (17, 18, 28, 29). The pregnant cervix undergoes four different phases: softening, ripening, dilation, and postpartum recovery (15, 18, 23). Softening begins soon after conception and progresses slowly. Ripening is a later phase near delivery that involves marked and accelerated softening (15, 18, 29).

In this study, the final marked ripening of the cervix in ewes on Day −1 was determined by quantifying the mean SWS values of the IOC. The evaluated mean SWS values of the IOC were similar at the evaluation points between Day −5 and Day −2. Starting the evaluation in the early weeks of pregnancy (12) and/or using wider intervals between evaluation points (14) may affect the detection of significant changes.

In this study, time-dependent differences in the mean SWS values of the EOC were not significant. This result showed that the IOC region better reflected the cervical changes during pregnancy and confirmed previous reports (11, 15). It has been reported that changes in SWS during pregnancy are more remarkable in the upper part of the cervix (15) and that the internal part shows a stronger relationship with gestational age (11). This finding, in which we did not find any time-dependent differences in the SWS values of the EOC during the prepartum period, is also consistent with our previous study (20). In that study, we evaluated the SWS values of the cervix in the postpartum period in ewes and found that the EOC values remained similar over time.

Peralta et al. (11) found that the external part of the cervix was significantly softer than the internal one. Ono et al. (15) reported that the lower part of the cervix had a lower SWS value than the upper part. In contrast to these previous reports (11, 15), the SWS values of the IOC and EOC determined in this study were similar, except on Day −1. The internal part of the cervix remains stiff for most of pregnancy to ensure gestational safety, but ripening occurs over the course of several days before parturition and involves marked and accelerated softening (15). In addition, cervical ripening has been reported to begin at the IOC (23) and to increase tissue homogeneity (30). We believe that the similar IOC and EOC values found in our study, in contrast to previous reports (11, 15), are due to our evaluations being conducted during the ripening period, close to parturition. Horinouchi et al. (12) reported higher SWS values for the IOC compared to the EOC in women at day 84 of pregnancy but found similar values at day 251. This study is consistent with the findings of Horinouchi et al. (12) in the later stage of pregnancy.

In this study, the mean SWS value of the IOC on Day 0 was higher than the values on Days −1, −2, and −3. The evaluation at Day 0 corresponded to the period of rapid cervical involution with marked contractions after lambing. This finding aligns with Carlson et al. (26), who found increased cervical SWS values during uterine contractions.

In this study, the thickness of the vulvar lips on Day −1 was similar to that on Day 0, but both were higher than the thickness measured on all other evaluation days. The mean SWS value of the vulvar lips on Day −1 was similar to that on Days −2 and 0, but it was lower than the value on Days −5, −4, and −3. In our previous study (20) on sheep during the postpartum period, we found that vulvar thickness on the first postpartum day was greater than the thickness measured at all subsequent evaluation points. In addition, it was found that the SWS value of the vulvar lips was lower on the 1st postpartum day compared to the 14th postpartum day and subsequent evaluation points. These values determined in the period close to parturition in both studies are compatible with each other. The vulva becomes progressively edematous, flaccid, and enlarged as parturition approaches, and these changes occur because of the changing hormonal milieu, including estrogen and relaxin (31). Five days before parturition, adrenocorticotropic hormone (ACTH) is secreted, stimulating the fetal adrenal glands to release cortisol. Cortisol, in turn, induces the enzyme 17α-hydroxylase, which shifts steroid production from progesterone to estrogen (32). Two days before parturition, an increase in the estrogen concentration occurs and continues right up to parturition (40–130 pmoL/mL) (33).

This study has potential limitations. First, our evaluations were conducted daily and limited to the period close to parturition. Evaluating cervical changes in sheep from conception to parturition could be more informative. Second, due to limited material, we were unable to determine a cutoff SWS value for the IOC that could be used as a predictor of parturition timing in ewes.

## Conclusion

Timely cervical remodeling is essential for successful parturition. Premature dilation of the cervix can lead to pregnancy loss, while inadequate ripening and dilation at term can lead to dystocia. Pregnancy monitoring in sheep helps reduce both maternal and lamb mortality rates. In this study, using modern ultrasonographic techniques, we were able to identify changes in the cervix and vulvar lips during the days preceding normal parturition and on the day of postpartum in ewes. Final ripening of the IOC and the edematous vulvar lips were indicated by low SWS values. Marked uterine contractions persisting during the postpartum period led to increased SWS values in the IOC.

## Data Availability

The original contributions presented in the study are included in the article/supplementary material, further inquiries can be directed to the corresponding author.
