# Correction: Shear wave speed changes in the cervix and vulvar lips of Kivircik ewes before and after parturition

**DOI:** 10.3389/fvets.2026.1814552

**Published:** 2026-02-27

**Authors:** Frontiers Production Office

**Affiliations:** Frontiers Media SA, Lausanne, Switzerland

**Keywords:** cervix, ewes, parturition, shear wave speed, vulvar lips

In the published article, incorrect Alt-text content for Figures 1–9 was used.

Alt-text content for Figures 1–9 has been updated as follows:

Figure 1: Panel A shows the placement of the transducer cranial to the mammary glands on the ventrolateral abdomen for the evaluation of cervical region of a sheep lying on its right side. Due to the advanced stage of pregnancy, we were able to visualize the cervix from this area after a gentle and short search. Panel B shows the placement of the transducer on a vulvar lip of a sheep.

Figure 2: Ultrasound image with three panels labeled A, B, and C. Panel A shows the reliability map. The measurements are performed in the area with green color, which indicates a high reliability (RLB index: 92%). Panel B shows the ROI positions placed near the IOC and quantitative evaluation of shear wave speed values. Also, a color scale (associated with the SWS values) which goes from dark blue to dark red is present. Panel C shows a color map of the area, which soft areas are revealed as blue.

Figure 3: Ultrasound image with three panels labeled A, B, and C. Panel A shows the reliability map. The measurements are performed in the area with green color, which indicates a high reliability (RLB index: 100%). Panel B shows the ROI positions placed near the EOC and quantitative evaluation of shear wave speed values. Also, a color scale (associated with the SWS values) which goes from dark blue to dark red is present. Panel C shows a color map of the area, which soft areas are revealed as blue.

Figure 4: Box plot showing cervix diameter (cm) from day minus five to day zero. Values correspond to the days before parturition (day−5 to day−1) and 6-12 hours postpartum (day 0) and are shown with boxes of different colors. Each box represents variability and mean values with letters above showing statistical significance comparisons.

Figure 5: Box plot showing shear wave speed (m/s) values of IOC from day minus five to day zero. Values correspond to the days before parturition (day−5 to day−1) and 6-12 hours postpartum (day 0) and are shown with boxes of different colors. Each box represents variability and mean values with letters above showing statistical significance comparisons.

Figure 6: Box plot showing shear wave speed (m/s) values of EOC from day minus five to day zero. Values correspond to the days before parturition (day−5 to day−1) and 6-12 hours postpartum (day 0) and are shown with boxes of different colors. Each box represents variability and mean values.

Figure 7: Ultrasound image with three panels labeled A, B, and C. Panel A shows the reliability map. The measurements are performed in the area with green color, which indicates a high reliability (RLB index: 100%). Panel B shows the ROI positions placed on a vulvar lip and quantitative evaluation of shear wave speed values. Also, a color scale (associated with the SWS values) which goes from dark blue to dark red is present. Panel C shows a color map of the area, which soft areas are revealed as blue.

Figure 8: Box plot showing vulvar lips thickness (cm) from day minus five to day zero. Values correspond to the days before parturition (day−5 to day−1) and 6-12 hours postpartum (day 0) and are shown with boxes of different colors. Each box represents variability and mean values with letters above showing statistical significance comparisons.

Figure 9 Alt: Box plot showing shear wave speed (m/s) values of vulvar lips from day minus five to day zero. Values correspond to the days before parturition (day−5 to day−1) and 6-12 hours postpartum (day 0) and are shown with boxes of different colors. Each box represents variability and mean values with letters above showing statistical significance comparisons.

The original version of this article has been updated.

